# Duplication of the right vertebral artery: MRA findings and review of the literature

**DOI:** 10.1186/s40064-016-2807-z

**Published:** 2016-07-19

**Authors:** Jiyeon Baik, Hye Jin Baek, Hwa Seon Shin, Kwang Ho Choi

**Affiliations:** Department of Radiology, Inje University College of Medicine, Haeundae Paik Hospital, 875 Haeundae-ro, Haeundae-gu, Busan, 48108 Republic of Korea; Department of Radiology, Gyeongsang National University School of Medicine and Gyeongsang National University Changwon Hospital, 11 Samjeongja-ro, Seongsan-gu, Changwon, 51472 Republic of Korea; Department of Radiology, Gyeongsang National University School of Medicine and Gyeongsang National University Hospital, 79 Gangnam-ro, Jinju, 52727 Republic of Korea; Department of Thoracic and Cardiovascular Surgery, Research Institute for Convergence of Biomedical Science and Technology, Pusan National University Yangsan Hospital, 20 Geumo-ro, Mulgeum-eup, Yangsan, 50612 Republic of Korea

**Keywords:** Duplication, Vertebral artery, Magnetic resonance angiography

## Abstract

**Introduction:**

Duplication of the vertebral artery (VA) is a rare vascular variant. To the best our knowledge, only fourteen cases have been reported with angiographic findings that they have dual origin of the VA from ipsilateral subclavian artery. Herein, we present a case of duplication of right VA which was incidentally detected by magnetic resonance (MR) angiography.

**Case description:**

A 69-year-old female patient presented with headache for 30 days. She underwent brain MR imaging with MR angiography for evaluating possible intracranial cause. There was a dual origin of the right vertebral artery (VA) as an incidental finding without other significant abnormalities.

**Discussion and Evaluation:**

Diagnosis of duplicated VA can be difficult due to its rarity and misinterpreted as the vascular dissection. In addition, a detailed knowledge of this variation is potentially important to prevent inadvertent challenges during endovascular procedure. Because duplicated VA has smaller lumen and usually enters the higher transverse foramen than those of normal side, it can be influence the choice or route of endovascular treatment.

**Conclusions:**

We suggested that the understanding of embryologic background about VA can be helpful to identify unexpected vascular findings on imaging studies in clinical practice.

## Background

Duplication of vertebral artery (VA) is rare and represents 0.72 % in autopsy studies (Bergman et al. 1988). This vascular variant is considered a developmental anomaly that shows a dual origin with a variable level of fusion in the neck (Goddard et al. 2001). According to previous articles, there are 36 reported cases about this vascular variant (Komiyama et al. 1999; Watanabe et al. 2015). However, a total of 14 cases have been reported with angiographic findings in literature that they have dual origin of the VA from ipsilateral subclavian artery (SCA), including a case of bilateral VA duplications. It is clinically significant because it can be mistaken for a dissection of VA (Thomas et al. 2008). Herein, we present a case of duplication of VA as an incidental finding during magnetic resonance angiography (MRA) examination of a patient with headache. Further, we also reviewed its embryological mechanism and clinical significance of this variation.

## Case description

A 69-year-old female patient presented with headache for 30 days. She had no other clinical manifestation or significant past medical history. There was no abnormal finding at the initial neurological examination.

**Fig. 1:**
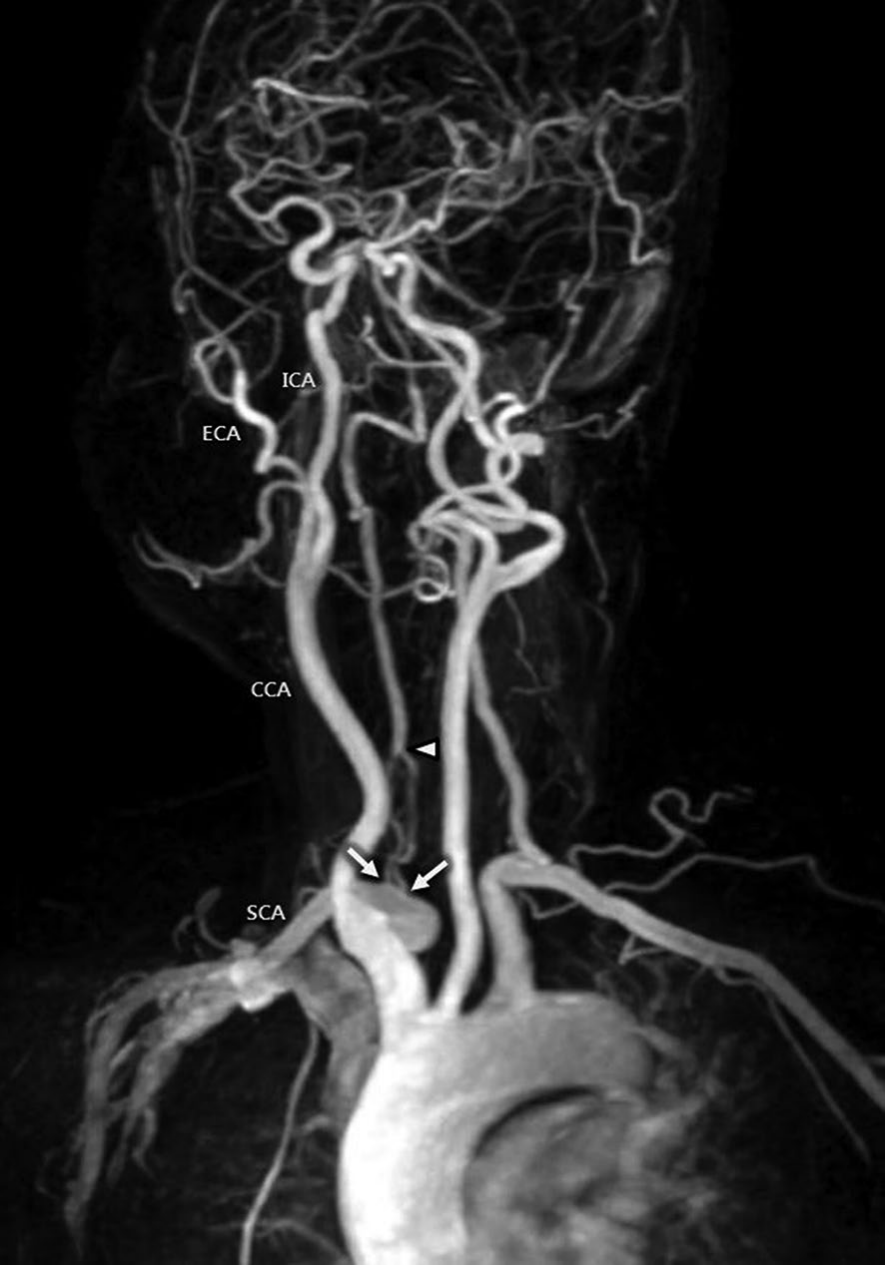
3D TOF MRA of duplicated right VA. 3D time-of-flight (TOF) magnetic resonance angiography (MRA) of left oblique view shows the duplicated ostia on the right subclavain artery (*arrows*). Two limbs of right vertebral artery (VA) united to form the distal part of the right VA *(arrowhead)*

She underwent cranial magnetic resonance image (MRI) with 3D time-of-flight (TOF) intracranial MRA and gadolinium-enhanced neck MRA, using 1.5T system (Signa Excite, GE Medical Systems, Milwaukee, WI, USA). Routine cranial MR images revealed no significant focal lesion in the brain parenchyma. 3D TOF intracranial MRA was also normal. Contrast-enhanced neck MRA demonstrated a duplication of the right VA with two origins from ipsilateral SCA before the origin of right internal thoracic artery (Fig. 1). The two limbs of duplicated VA confined to proximal V1 segment, and converged to form one main trunk at the 3.6 cm distal from each orifice. The first and second limbs of duplicated V1 segment measured 2.8 and 2.6 mm in diameter at the points of origin, respectively. The distance between two limbs calculated 8.0 mm. The diameter of the common trunk of right VA was 3.8 mm at the point of reconstitution. On the left side, the VA arose from the left SCA and measured 4.1 mm in diameter. There was no other vascular abnormality in the major cervical arteries. The patient was managed conservatively and the patient’s clinical symptoms were relieved after 7 days.

## Discussion

Duplicated VA is a rare vascular variant (Bergman et al. 1988). It is defined that VA has two origins with fusion at a variable level of the neck. Duplication of the VA has been confused with fenestration of VA, which is a different condition showing a single origin with a short segment of two limbs of VA and one connection with each other along its course (Goddard et al. 2001; Thomas et al. 2008).

Embryologically, the VA is formed by the development of postcostal longitudinal anastomosis which links the intersegmental arteries. The intersegmental arteries eventually obliterated with an exception of the seventh, which becomes the proximal SCA and includes the VA point of origin in adults (Newton and Mani 1974). Duplication of VA is a result from abnormality of embryologic development of aortic arch and persistence of the intersegmental artery (Polguj et al. 2013a). Whereas dual origin of VA originating from the right SCA or brachiocephalic artery occurs due to persistence of the right fourth or fifth (or less frequently third) intersegmental arteries, persistence of the left fourth or fifth (or less frequently third) intersegmental arteries can result in aortic origin or dual origin of the left VA (Komiyama et al. 1999). In addition, the level of entrance into the transverse foramen indicates which intersegmental artery or arteries persist (Meila et al. 2012).

To our best knowledge, only fourteen cases have been reported the duplicated VA with dual origin from SCA among total 36 reported cases of duplicated VA (Table 1) (Babin and Haller 1974; Harada et al. 1987; Hashimoto et al. 1987; Mahmutyazicioğlu et al. 1998; Goddard et al. 2001; Ionete and Omojola 2006; Harnier et al. 2008; Mordasisni et al. 2008; Thomas et al. 2008; Meila et al. 2012; Melki et al. 2012; Polguj et al. 2013b; Rameshbabu et al. 2014). Unilateral duplication of VA was more commonly observed in left side (Goddard et al. 2001). In most cases, the two limbs of the duplicated VA arise from the aorta and the SCA. The limb of duplicated artery also can be originated from common carotid artery, brachiocephalic trunk, or thyrocervical trunk (Bergman et al. 1988; Rameshbabu et al. 2014). Generally, the normal VA is the 1st vessel branching of the SCA and it contributes to the posterior circulation of the head and neck. Whereas normal VA almost always enters the C6 transverse foramen, duplication of VA usually enters the higher transverse foramen (Thomas et al. 2008).

**Table 1:** Reports of duplicated origin of vertebral artery from the subclavian artery proved by imaging modality

No	Age	Sex	Clinical symptom	Underlying disease	Side	Level of fusion	Accompanying anomalies	Imaging modality	Reference	Published
1	18	F	Cervicooccipital pain	Epilepsy	Right	C5	Dolichoarterial loop of the left VA	Conventional angiography	Babin and Haller	1974
2	67	M	Dizziness, slight left mortor weakness	None	Right	C5	None	Contrast-enhanced CT	Hashimoto et al.	1987
3	70	M	Head heaviness, dizziness	None	Right	C4	Hypoplastic left VA	Conventional angiography	Harada et al.	1987
4	62	M	Vertigo, weakness, nausea	Thrombosis at the origin of duplicated artery	Left	Higher than C2	None	Color Doppler ultrasonograrphy, CT angiography, MR angiography	Mahmutyazicioglu et al.	1998
5	66	F	Dysarthria, left hemisensory disturbance and hemiplegia	Acute right cerebral infarction, hypertension, hypercholesterolaemia	Right	Level of carotid bifurcation	None	MR angiography	Goddard et al.	2001
6	83	M	Mild cognitive impairment	None	Both	Right: C4–5 Left: C5–6	None	MR angiography	Ionete and Omojola	2006
7	49	F	None	None	Right	C6	Unruptured midbasilar trunk aneurysm	Conventional angiography	Thomas et al.	2008
8	61	F	Dizziness	None	Right	Not mentioned	Duplicated right CCA, Fenestration of left CCA	MR angiography	Harnier et al.	2008
9	48	M	Not mentioned	Recent middle cerebral artery stroke	Right	C4/5	Duplicated left VA (originated from aortic arch and left subclavian artery), left internal carotid artery stenosis	MR angiography	Mordasisni et al.	2008
10	54	F	Not mentioned	Transient ischemic attack	Right	C4	Thyrovertebral trunk	CT angiography	Meila et al.	2012
11	43	F	Severe headache	Mild subarachnoid hemorrhage	Right	C4	Duplicated left VA (originated from aortic arch), Thyrovertebral trunk, Intracranial aneurysm	CT angiography	Meila et al.	2012
12	51	M	Acute vertigo	Recent infarction of right cerebellar vermis, Dissection on right duplicated vertebral artery	Right	V2 segment	None	CT angiography, MR angiography	Melki et al.	2012
13	43	M	Headache, left limb weakness	Right ICA dissection, Ehlers–Danlos syndrome	Left	C5–6	None	CT angiography, color Doppler ultrasonograrphy	Polguj et al.	2013a, b
14	36	M	Dizziness	None	Right	C4–5	Duplicated left VA (originated from aortic arch and left subclavian artery)	CT angiography	Rameshbabu et al.	2014

The clinical feature of the duplication of the VA is nonspecific and usually asymptomatic. And it is still controversy whether duplication of the VA is associated with other pathologic conditions. Ionete and Omojola (2006) demonstrated that duplicated VA was normal variation as incidental finding with no significant clinical or pathologic consequences. Conversely, anecdotal reports stated this vascular variant may predispose the patient to VA dissection, intracranial aneurysm, kinking, and arteriovenous malformation (Rameshbabu et al. 2014).

Dual origin of the VA is usually diagnosed as an incidental finding that occurs during imaging workups for other clinical situations. However, a detailed knowledge of this variation is potentially important to prevent inadvertent diagnostic or therapeutic challenges during endovascular procedure. Because duplicated VA has smaller lumen of its proximal V1 than that of normal side, it can be influence the choice or route of endovascular treatment (Thomas et al. 2008; Ionete and Omojola 2006).

## Conclusion

In conclusion, we provide a rare case of the right VA with dual origin as an incidental finding on MRA. This report suggested that the awareness of embryologic background about VA can be helpful to identify unexpected vascular findings on computed tomographic angiography (CTA) or MRA, and differentiate this variation from other pathologic conditions such arterial dissection in clinical practice.

## Abbreviations

VA: vertebral artery
SCA: subclavian artery
MRA: magnetic resonance angiography
MRI: magnetic resonance image
TOF: time-of-flight
CTA: computed tomographic angiography
